# Supplementary material for: Multiplexed expansion revealing for imaging multiprotein nanostructures in healthy and diseased brain
Source: Nat Commun. 2024 Nov 9;15:9722. doi: 10.1038/s41467-024-53729-w (PMC11550395; doi:10.1038/s41467-024-53729-w)
Supplement: Supplementary file 1 — Supplementary Information [file 41467_2024_53729_MOESM1_ESM.pdf]

Supplementary Information

Multiplexed expansion revealing for imaging multiprotein nanostructures in healthy and diseased brain

Jinyoung Kang[1,2,*], Margaret E. Schroeder[1,3,*], Youngmi Lee[1], Chaitanya Kapoor[4], Eunah Yu[1], Tyler B. Tarr[5], Kat Titterton[1], Menglong Zeng[1], Demian Park[1], Emily Niederst[6], Donglai Wei[7], Guoping Feng[1,2,3,8], Edward S. Boyden[1,2,3,9,10,11,12,13**]

Contents

Supplementary Figures

Supplementary Figure 1. Guidelines for choosing multiExR reference channel(s) and registration method(s).

Supplementary Figure 2. Primary validation of stripping and re-staining efficiency with multiExR.

Supplementary Figure 3. Additional validation of stripping and re-staining efficiency with multiExR.

Supplementary Figure 4. 10-plexed nanoscale characterization of putative synapses in cultured neurons.

Supplementary Figure 5. A single z-plane of synaptic proteins showing colocalization of VGlut1, Bassoon, and SynGAP.

Supplementary Figure 6. Example use cases of additional registration algorithms.

Supplementary Figure 7. Masking can improve registration error.

Supplementary Figure 8. Sensitivity of results to median and size filter sizes.

Supplementary Tables 1-19

Supplementary Notes 1-4

Supplementary References

A. multiExR experiment planning decision tree

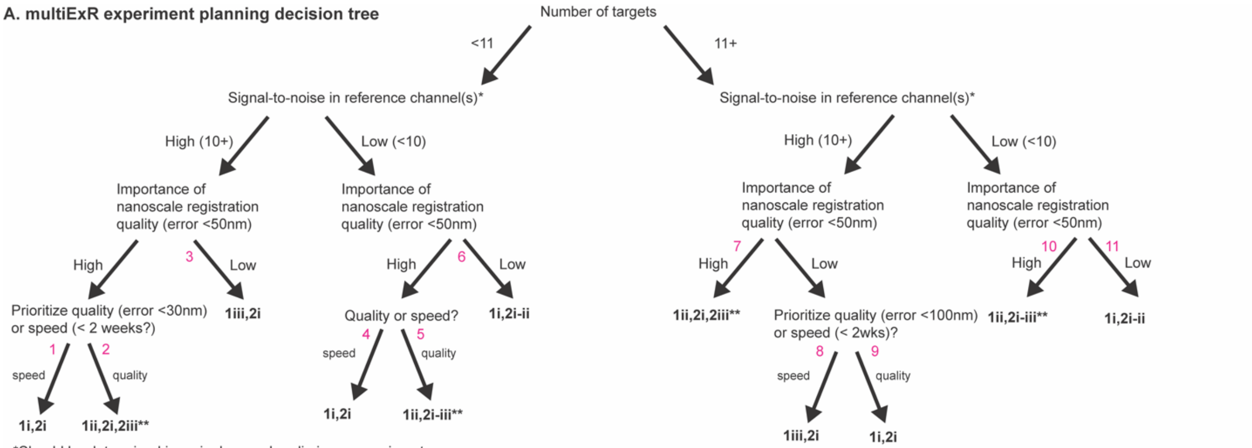

*Should be determined in a single-round preliminary experiment.

If registering synaptic proteins with maximum accuracy is important, and a synaptic scaffold protein has been imaged in each round (see **C(2)iii).

B. Simplified table for choosing reference channels

	i	ii	iii
# target proteins/round	2	1-2	3
average registration error	20-70nm	10-40nm	50-100nm
channel wavelengths (nm)	488/546: target proteins<br>633: multi-target reference	488 and/or 546: target protein(s),<br>594 Lectin + 633: synaptic protein reference	488/546/633: target proteins,<br>594 Lectin: reference

C. Legend and details

1. Reference channel

i. 3+ proteins in one reference channel (Figs. 3-5, Extended Data Figs. 2-4)
PROS: improved registration relative to (iii), usually in the 20-70nm range, more targets per round than (ii)
CONS: lower registration quality relative to (ii), 2 target proteins per round (multi-target reference cannot be in 594 nm channel, as bleedthrough is likely to overlap with target proteins in 546 and 633 nm channels)

ii. Multiple single-protein channels combined (normalized and summed) to a single reference channel (Fig. 2)
PROS: in our hands, consistently improved registration relative to (i), usually in the 10-40nm range, because relative intensities of each reference protein channel can be normalized
CONS: only 1-2 target channels can be imaged per round

iii. One single-protein as a single reference channel (e.g., Lectin stain in 594nm, used in earlier datasets not shown)
PROS: can allow 3 targets to be imaged per round (reference channel in 594nm, targets in 488nm, 546nm, and 633nm), if bleedthrough is unimportant (e.g., synaptic proteins being imaged with blood vessel as main feature in registration channel)
CONS: lower registration quality (usually in the 50-100nm range) and potential failures if reference channel protein is not dense (>3% of field of view by volume for a ~20um thick volume in physical units), bright (SNR > 10), and lacks features at multiple scales (by qualitative inspection)

2. Registration algorithm

i. Global feature-based (ExSeq Processing, Alon et al., Science 2021; Figs. 2-4, Extended Data Figs. 2-4)
PROS: fast (with GPU), and usually accurate within 50nm for reference channels with high signal-to-noise
CONS: slower (without GPU), high failure rate (>50%) for noisy reference channels (SNR < 10) if strategy 1(iii) is chosen

ii. Global intensity-based (Elastix, Klein et al., 2010; Extended Data Fig. 2, Fig. 5, Extended Data Fig. 4)
PROS: Can outperform (i) in cases where there is low staining intensity (SNR < 10)
CONS: Cannot run on GPU, subpar results when there are multiple structures e.g., neuronal processes due to global affine transformation, needs manual setting of multiple filtering parameters in case of noisy images

iii. Local point-based (Landmark-based RBF, Rohr et al., 2003; Extended Data Fig. 4)
PROS: capable of aligning "fine" structures e.g., synapses by computing a non-linear deformation field; can improve local registration quality of small, punctate nanostructures like synapses beyond (i) and (ii)
CONS: need to generate dense deformation field (same resolution as original image; high memory usage), cannot run on GPU, requires consistent nanostructure signal in each imaging round (e.g., similar synaptic markers), local deformation may disrupt global registration quality

Supplementary Figure 1. Guidelines for choosing multiExR reference channel(s) and registration method(s). A, Decision tree to help experimenters select a starting point for reference channel and registration algorithm based on experimental goals and practical constraints. Note that these choices can be further optimized based on the results of pilot experiments. Magenta numbers denote paths on the tree for easy reference in the text. Magenta numbers indicate paths on the decision tree, as referred to in the main text. **B**, Simplified table for choosing reference and registration channels. Average registration error ranges are an estimate. **I-iii** refer to the reference channel options detailed in **C**. **C**, Legend and details for (**A**) and (**B**), detailing the pros and cons of each (1) reference channel option and (2) registration algorithm option based on our experience. We do not provide a blanket recommended sequence for the staining of different targets. The prioritizing of protein targets in rounds may depend on the purpose of the study, the expression level of the protein targets, and the quality and host species of the antibodies. It may be helpful to image one or more structural/marker proteins in the earlier rounds to choose the most appropriate fields of view for imaging in later rounds. Additionally, low-expressing protein targets and/or

antibodies that yield low signal to noise might be better suited for earlier rounds, where the chance of signal degradation is lowest (although note the observation, in the main text, that some epitopes may benefit from an antigen retrieval effect of stripping), while high-expressing protein targets and/or antibodies that yield high signal to noise might be better suited for later rounds. Abbreviations: SNR, signal to noise ratio; GPU, graphics processing unit; RBF, radial basis function.

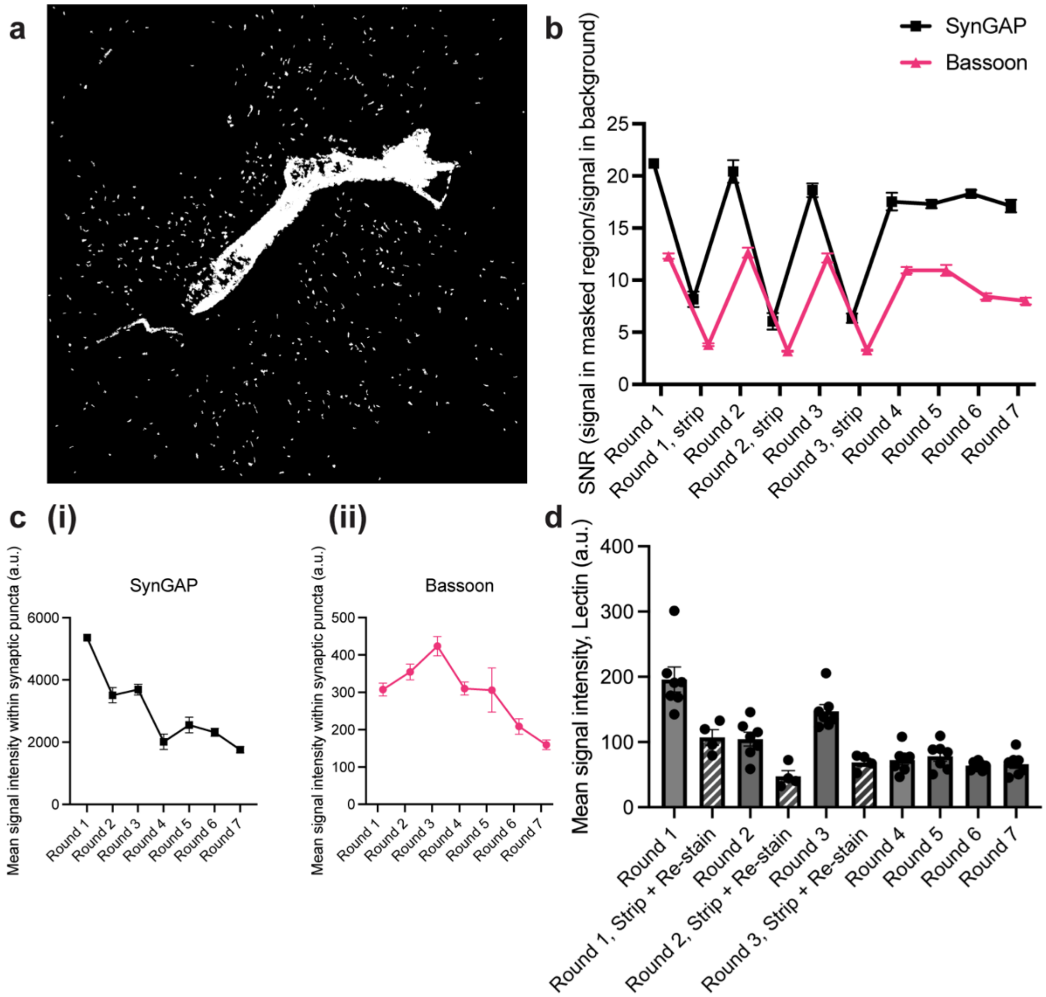

Supplementary Figure 2. Primary validation of stripping and re-staining efficiency with multiExR. a, The summed reference channel mask for **Fig. 2**, used to calculate the number of objects detected in the whole field of view. **b,** Signal-to-noise ratio, calculated as signal in masked region divided by signal in background, see **Methods**) of synaptic proteins within manually-identified synaptic ROIs for the primary validation dataset (n = 7 fields of view from one mouse, mean is taken over 51-53 ROIs per field of view). **c**, Mean signal intensity within synaptic puncta (masked) within manually-identified synaptic ROIs for the primary validation dataset for (i) SynGAP and (ii) Bassoon channels (n = 7 fields of view from one mouse, mean is taken over 51-53 ROIs per field of view). **d,** Mean fluorescence signal intensity in arbitrary units (a.u.) of the Lectin channel after several rounds of staining, stripping and re-staining (n = 7 fields of view from one mouse, mean is taken across the entire field of view). For population and comparative statistics, see **Supplementary Table 3.** Source data are provided as a Source Data file.

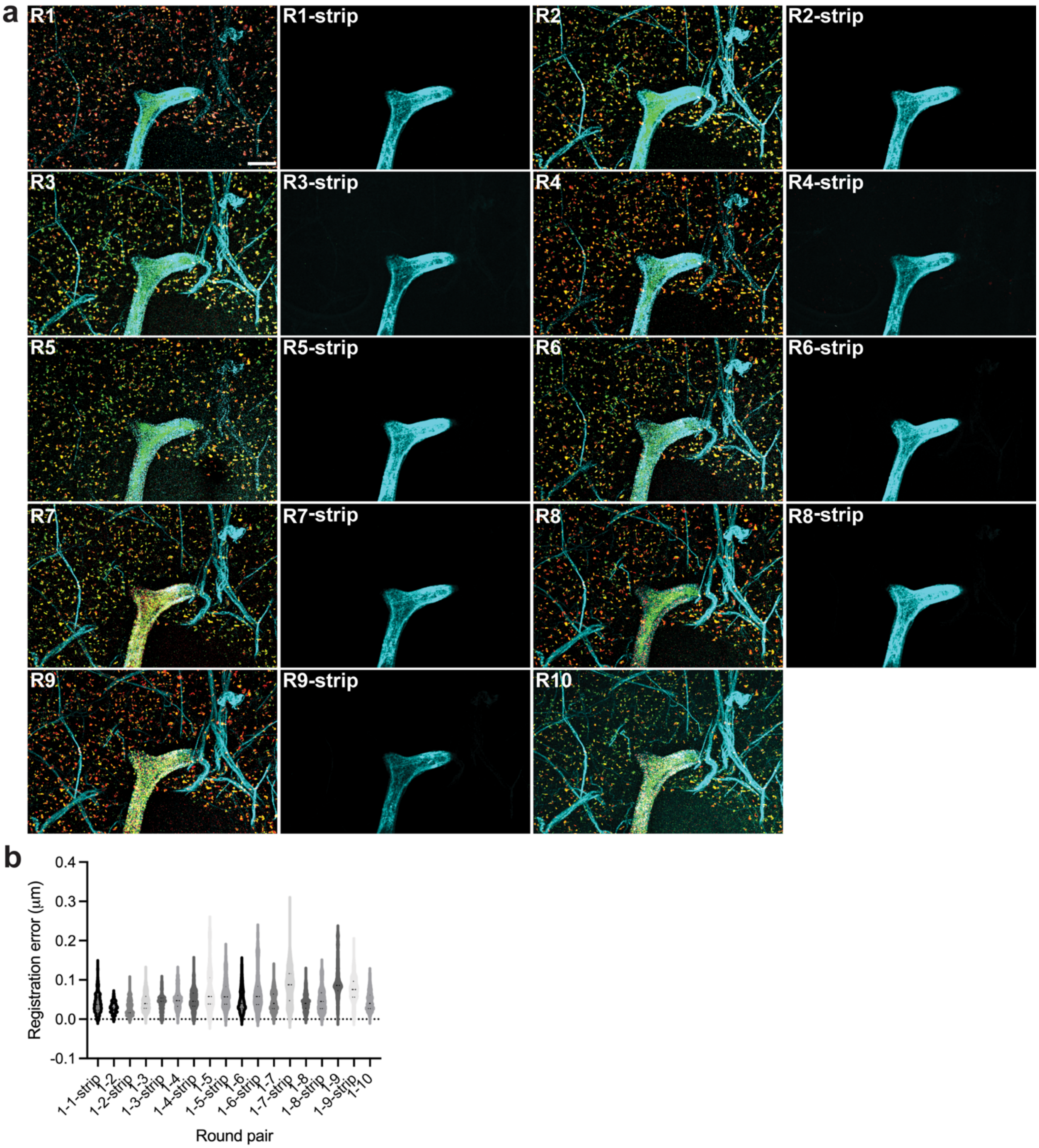

Supplementary Figure 3. Additional validation of stripping and re-staining efficiency with multiExR. a, Cropped maximum intensity projection for an example field of view from the registered secondary validation dataset, showing rounds 1-10 of staining and accompanying stripping rounds (red: SynGAP, green: NMDAR1, cyan: Lectin/SMI/GFAP/Homer reference channel). Pixel intensities are adjusted to the same minimum and maximum value for pairs of stripping and staining rounds. However, the min-max range is set differently for staining rounds imaged using different microscopes. Scale bar, 2 µm in biological units. **b**, Estimated population distribution (violin plot of density, with a dashed line at the median and dotted lines at the quartiles) of the registration error in an exemplary field of view from the secondary validation dataset (**a**), with the 95% confidence interval for each round pair tabulated below

(n = 924-990 randomly sampled subvolumes from one field of view from one mouse, see **Supplementary Table 4** for complete statistics). Source data are provided as a Source Data file.

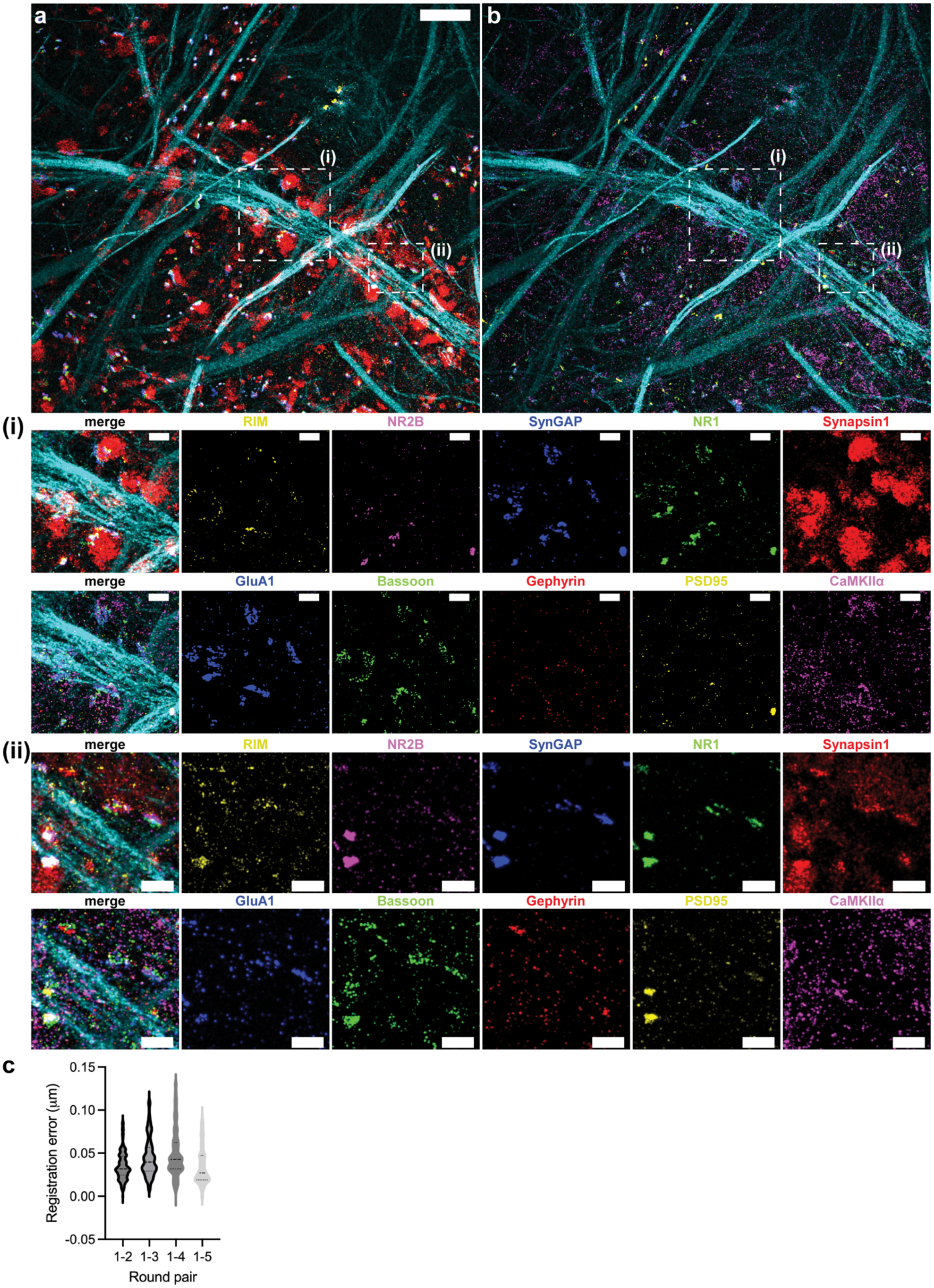

	1-2	1-3	1-4	1-5
Lower 95% CI of mean	0.03392	0.04364	0.04809	0.03304
Upper 95% CI of mean	0.03598	0.04645	0.05156	0.03566

Supplementary Figure 4. 10-plexed nanoscale characterization of putative synapses in cultured neurons. **a-b,** Composite 5-channel maximum intensity projection of a representative field of view of synaptic proteins in cultured hippocampal neurons obtained using multiExR. Scale bar, 2 μm in biological units. **a(i)-b(ii),** Single-channel and composite maximum intensity projections of synaptic proteins in the boxed regions from **(a)** and **(b)**. Scale bar, 500 nm in biological units **c,** Estimated population distribution (violin plot of density, with a dashed line at the median and dotted lines at the quartiles) of the registration error in a representative field of view, with the 95% confidence interval for each round pair tabulated below (see **Methods**, n = 870-1,000 randomly sampled subvolumes from one field of view from one batch of cultured neurons, see **Supplementary Table 13** for full statistics). Source data are provided as a Source Data file. This experiment was performed a single time and not repeated.

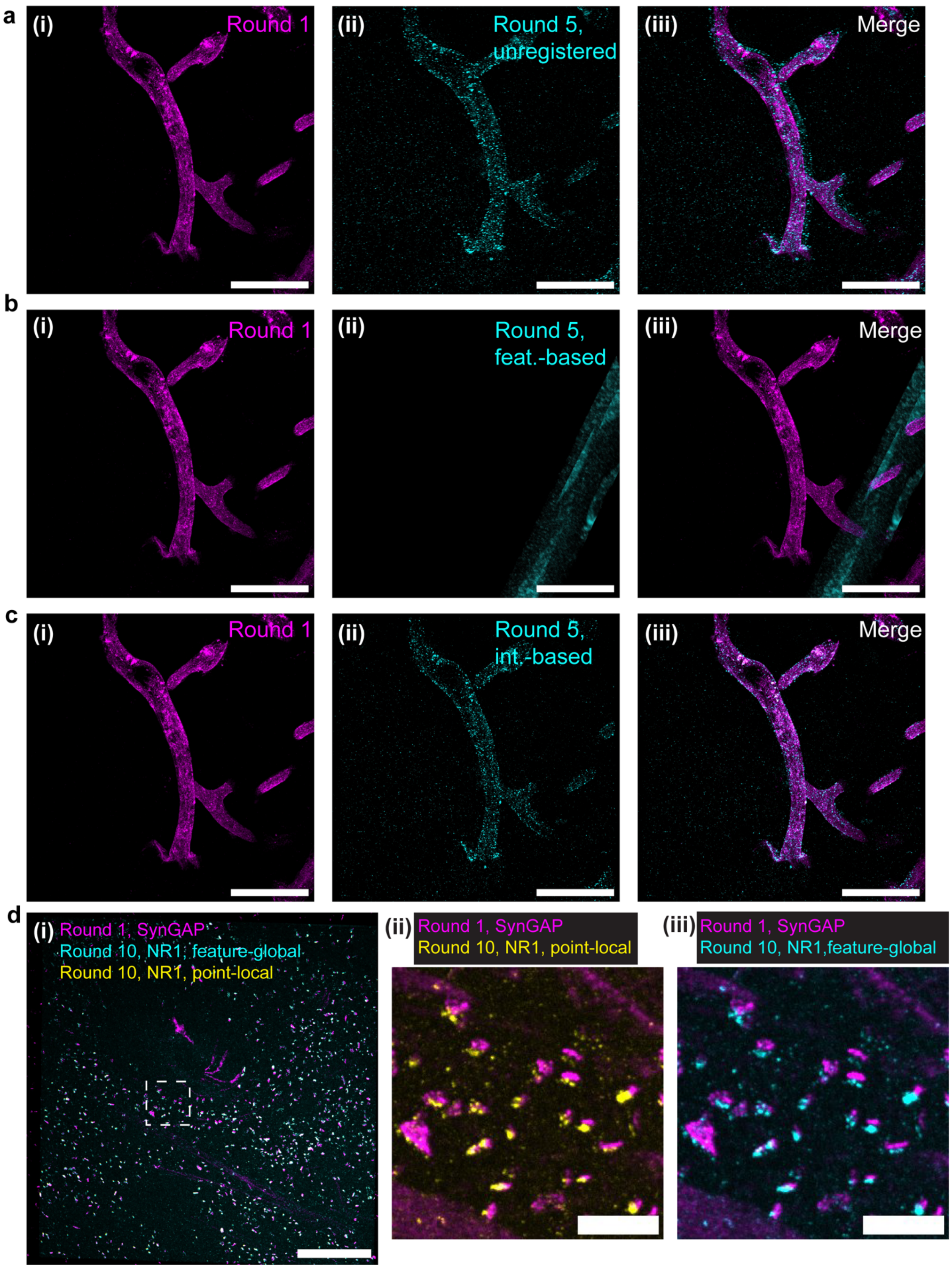
a
(i)
Round 1
(ii)
Round 5,
unregistered
(iii)
Merge
b
(i)
Round 1
(ii)
Round 5,
feat.-based
(iii)
Merge
c
(i)
Round 1
(ii)
Round 5,
int.-based
(iii)
Merge
d
(i)
Round 1, SynGAP
Round 10, NR1, feature-global
Round 10, NR1, point-local
(ii)
Round 1, SynGAP
Round 10, NR1, point-local
(iii)
Round 1, SynGAP
Round 10, NR1,feature-global

Supplementary Figure 5. Example use cases of additional registration algorithms. **a(i)**, Maximum intensity projection (MIP) of first-round reference channel (Lectin) in the cortex of the 5xFAD mouse brain. **a(ii)**, MIP of the fifth-round reference channel (Lectin/SMI/GFAP), without registration. **a(iii)** Merged overlay of **(i)** and (**iii**). **b,** Same as **(a)**, for the fifth-round image volume registered using the global feature-based ExSeqProcessing registration algorithm (algorithm **2(i)** in **Supplementary Fig. 1C**). **c,** Same as **(a)**, for the fifth-round image volume registered using the global intensity-based registration algorithm (algorithm **2(ii)** in **Supplementary Fig. 1C**). **d(i),** MIP composite overlay of a first-round synaptic channel (SynGAP), a tenth-round synaptic channel (NR1) registered using the global feature-based ExSeqProcessing registration algorithm, and a tenth-round synaptic channel (NR1) registered using the global intensity-based algorithm (algorithm **2(ii)**) followed by the local point-based registration algorithm **2(iii)** in **Supplementary Fig. 1C**). **d(ii)** Zoom-in of the boxed region in **d(i)** showing the composite overlay of the first-round and tenth-round synaptic channels from the point-based local registration algorithm (algorithms **2(ii)** and **2(iii)** in **Supplementary Fig. 1C**). **d(iii)** Same as **d(ii)**, for the global feature-based ExSeqProcessing registration method (algorithm **2(i)** in **Supplementary Fig. 1C**).

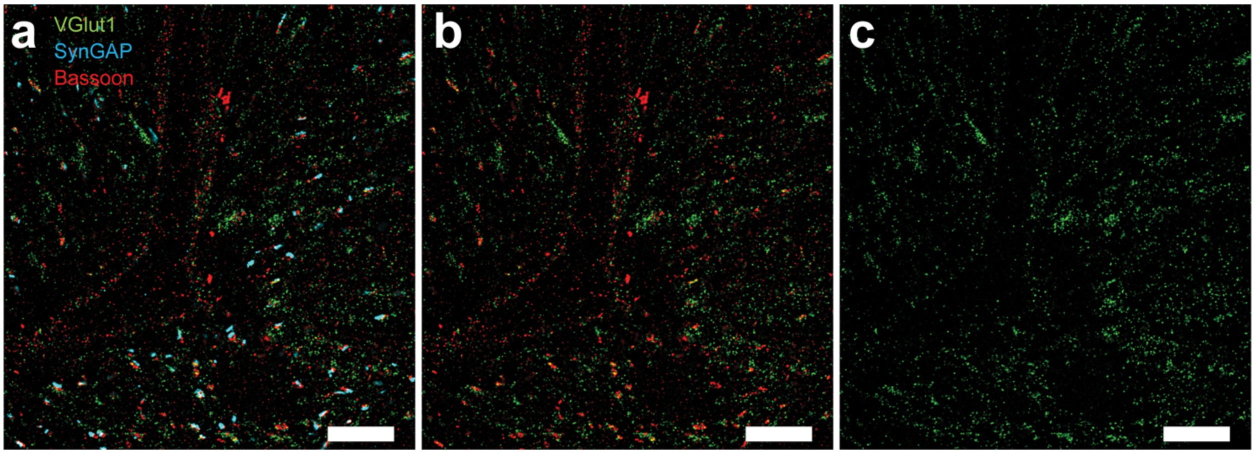

Supplementary Figure 6. A single z-plane of synaptic proteins showing colocalization of VGlut1, Bassoon, and SynGAP. Composite **(a)** 3-channel, **(b)** 2-channel, and **(c)** single channel of a representative field of view showing synaptic proteins in mouse somatosensory cortex obtained using multiExR (from one of two mice from one batch of experiments). Scale bar, 2 μm in biological units.

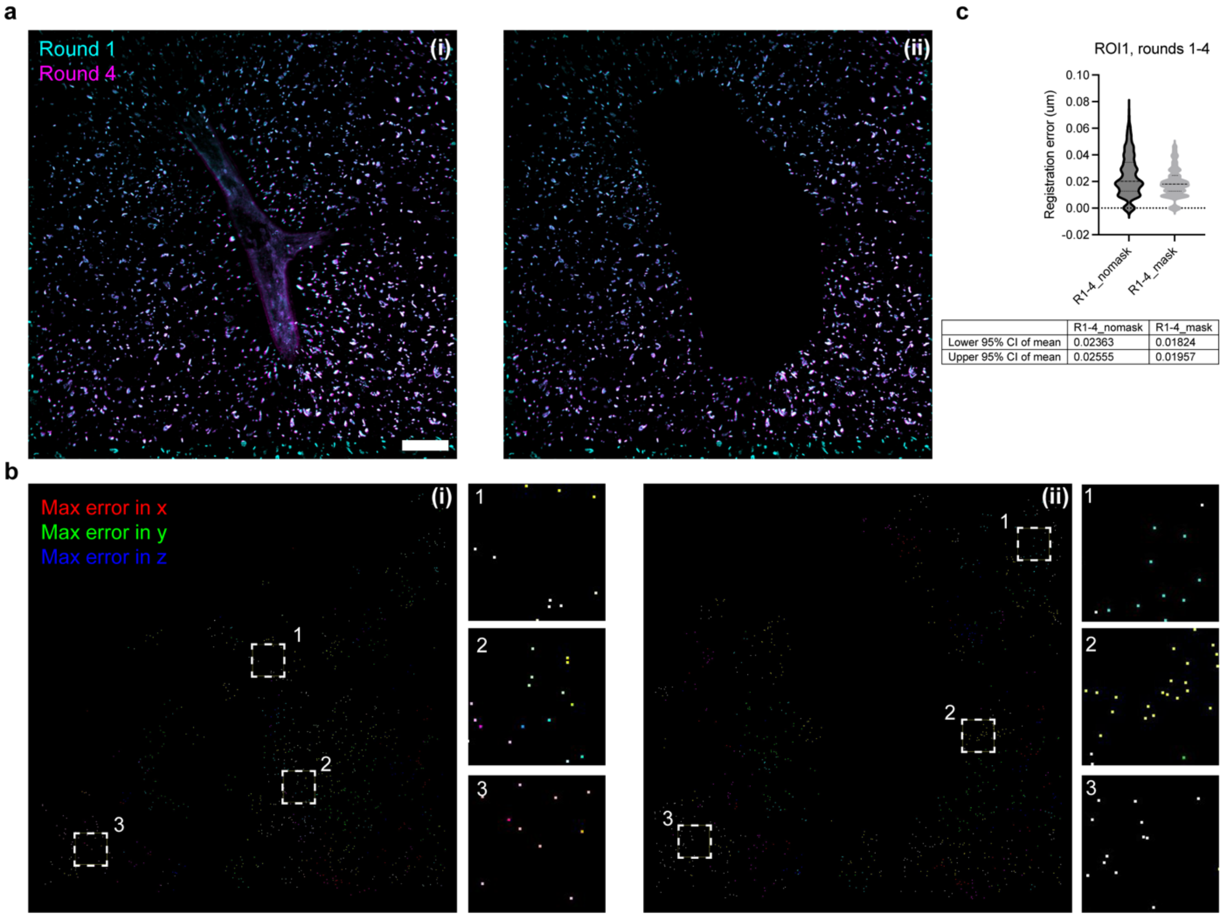

Supplementary Figure 7. Masking can improve registration error. a(i), Composite maximum intensity projection of the synGAP channel in imaging rounds 1 and 4 of the primary validation dataset, for the entire field of view whose registration error is shown in **Fig. 2f** (ROI1). **(ii)** Same as **(i)**, with a visually-determined higher-registration error area masked out. The mask was created and applied manually in Fiji using image math. **b(i),** RGB format maximum intensity projection of the same field of view as in **(a)**, with 100-voxel subvolumes colored by the maximum registration error (normalized to image maximum of 255) in x (red), y (green) and z (blue). To assist in visualization, the image was rescaled from 0 (minimum) to 30 (maximum) intensity, so that 30 became 255. **1-3)** Zoomed insets of the boxed regions in **(i)**. **(ii)** Same as **(i)**, with the higher-registration error area masked out. We qualitatively examined both the composite overlay of the synGAP channel (used to calculate registration error) and the RGB image showing the magnitude of the registration error in x, y, and z in red, green, and blue to identify the area of higher registration error in the x-y plane (note that the z-stack was already trimmed to the middle region, removing edges that are prone to registration offsets, see **Methods**). **c,** Estimated population distribution (violin plot of density, with a dashed line at the median and dotted lines at the quartiles) of the registration error in the unmasked, original field of view (dark gray) or the masked field of view (light gray), after outlier removal as described in the **Methods**. The 95% confidence interval of the mean registration error is provided below (n = 988 subvolumes for unmasked image after outlier removal, n = 966 subvolumes for masked image after outlier removal). Using this procedure, we were able to reduce the 95% confidence interval of the mean registration error (across subvolumes within each field of view) from 23.63-25.55nm to 18.24-19.57nm. Of note, the masked field of view retains ~80% of the original area in the x-y plane. In theory, the registration error range could be reduced even further by more aggressive cropping to even lower registration-error regions. Source data are provided as a Source Data file.

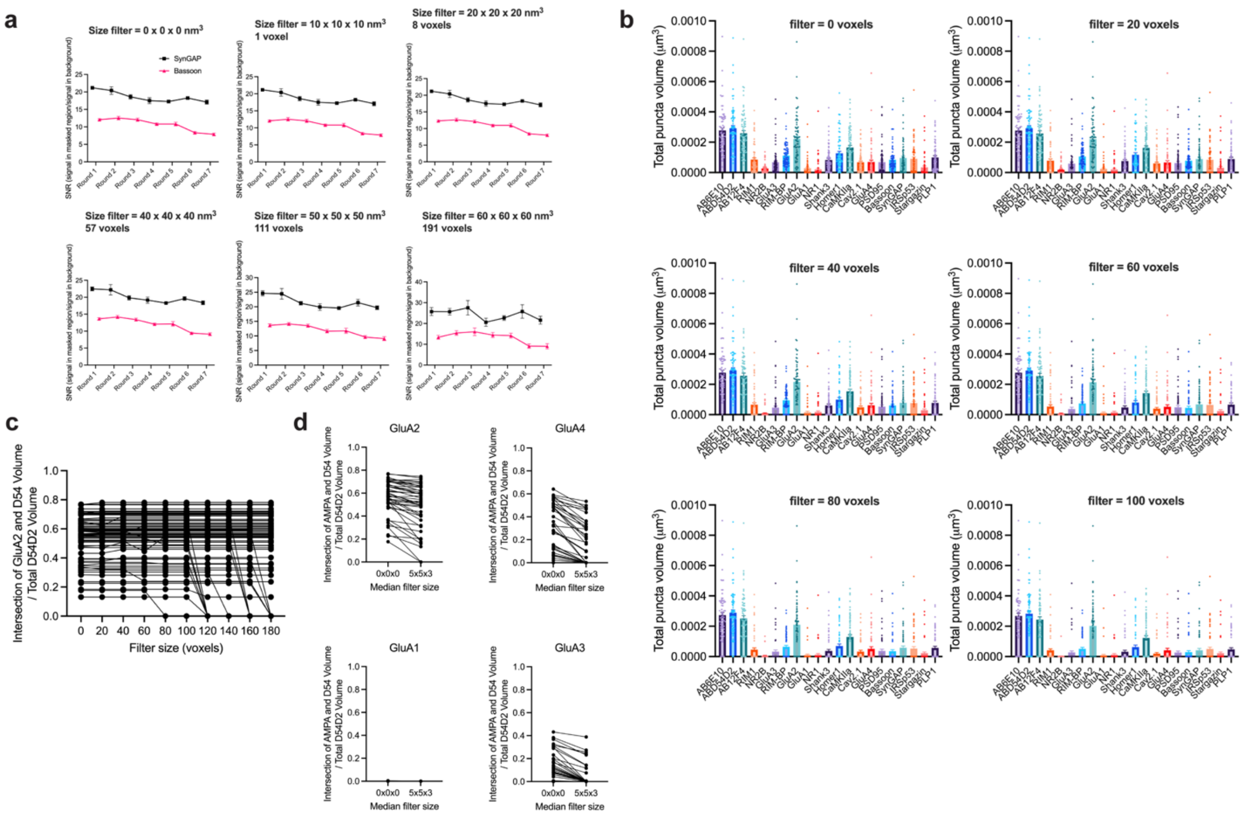

Supplementary Figure 8. Sensitivity of results to median and size filter sizes. **a,** Signal-to-noise ratio, calculated as signal in masked region divided by signal in background (as in **Supplementary Fig. 2b**) of synaptic proteins within manually-identified synaptic ROIs for the primary validation dataset with various minimum size filters (n = 7 fields of view from one mouse, mean is taken over 51-53 ROIs per field of view). **b,** Bar plots of total volume of select proteins within Aβ nanocluster ROIs (as in **Fig. 4b**, n = 71 ROIs from 9 fields of view from 2 5xFAD animals; error bars indicate mean +/- standard error of the mean), with various minimum size filters. **c,** Fraction of volume of D54D2 occupied by AMPA receptor, as in **Fig. 4c,** as a function of minimum filter size, where data points from the same nanocluster ROI are connected by lines (n = 44 nanocluster ROIs from 8 fields of view from 2 5xFAD animals). **d**, Same as **(c)**, but as a function of median filter size. Source data are provided as a Source Data file.

Supplementary Table 1. Mean feature density of masked binary reference channel (summed mask of each individual channel, which was used for registering this dataset), measured by volume, across 7 fields of view and 7 rounds in the primary validation dataset. Total feature volume is calculated as the sum of all nonzero pixels in the binary image (see **Methods**) across all channels. Largest feature volume is calculated as the mean number of nonzero pixels in the largest connected component across all channels, and smallest feature volume is calculated as the mean number of nonzero pixels in the smallest connected component across all channels. The mean was first taken across all rounds for each field of view, and then across all fields of view.

	Total feature volume / total image volume	Largest feature volume / total feature volume	Smallest feature volume / total feature volume
Mean	0.01256	0.4921	2.832×10^{-5}
95% CI	[0.01110,0.01402]	[0.3707,0.6136]	[2.368×10^{-5}, 3.297×10^{-5}]

Supplementary Table 2. Registration error for the primary validation dataset, measured using the SynGAP channel for the staining rounds, which was chosen based on its high signal-to-noise ratio across rounds, and the Lectin channel for the stripping rounds, which were re-stained for Lectin (**Fig. 2, Supplementary Fig. 2**).

**Field of view name**	**Round pair**	**# of ROIs after outlier removal**	**Mean (um)**	**Lower 95% of mean (um)**	**Upper 95% of mean (um)**
ROI1	1-2	989	0.01522	0.01467	0.01578
ROI1	1-3	995	0.02351	0.02271	0.0243
ROI1	1-4	990	0.02539	0.02443	0.02635
ROI1	1-5	972	0.02427	0.02337	0.02516
ROI1	1-6	951	0.02586	0.02491	0.02681
ROI1	1-7	974	0.02756	0.02657	0.02855
ROI2	1-2	985	0.01397	0.01342	0.01452
ROI2	1-3	993	0.02931	0.02835	0.03027
ROI2	1-4	945	0.0189	0.01822	0.01958
ROI2	1-5	970	0.02005	0.01916	0.02094
ROI2	1-6	937	0.02105	0.02017	0.02194
ROI2	1-7	921	0.02091	0.01999	0.02183
ROI3	1-2	1000	0.0323	0.03109	0.03351

ROI3	1-3	1000	0.04885	0.04708	0.05062
ROI3	1-4	993	0.05775	0.05543	0.06007
ROI3	1-5	992	0.05781	0.0559	0.05971
ROI3	1-6	972	0.07241	0.06934	0.07548
ROI3	1-7	959	0.07244	0.06937	0.07552
ROI4	1-2	911	0.09789	0.0938	0.102
ROI4	1-3	964	0.0176	0.01696	0.01825
ROI4	1-4	943	0.02024	0.01929	0.0212
ROI4	1-5	963	0.02192	0.02087	0.02296
ROI4	1-6	940	0.02061	0.01966	0.02157
ROI4	1-7	909	0.02081	0.02001	0.02162
ROI5	1-2	977	0.01802	0.01746	0.01857
ROI5	1-3	980	0.03114	0.02995	0.03233
ROI5	1-4	932	0.02154	0.02078	0.02231
ROI5	1-5	945	0.02512	0.02434	0.02589
ROI5	1-6	944	0.02584	0.02489	0.02679
ROI5	1-7	932	0.02733	0.02641	0.02825
ROI6	1-2	999	0.01785	0.01738	0.01833
ROI6	1-3	945	0.02045	0.01976	0.02114
ROI6	1-4	938	0.02037	0.01972	0.02102
ROI6	1-5	947	0.02624	0.02543	0.02705
ROI6	1-6	967	0.02613	0.02525	0.02701
ROI6	1-7	878	0.01903	0.0184	0.01967
ROI8	1-2	939	0.02022	0.01934	0.02109
ROI8	1-3	991	0.03711	0.03568	0.03855
ROI8	1-4	799	0.02052	0.0198	0.02123

ROI8	1-5	1000	0.03439	0.03348	0.0353
ROI8	1-6	977	0.05826	0.05597	0.06054
ROI8	1-7	997	0.06845	0.06556	0.07134
ROI3	1-1_strip	1000	0.04976	0.04842	0.05109
ROI3	1-2_strip	1000	0.05802	0.05623	0.05981
ROI3	1-3_strip	1000	0.07903	0.07684	0.08122
ROI4	1-1_strip	1000	0.01805	0.01731	0.01879
ROI4	1-2_strip	1000	0.03002	0.02882	0.03121
ROI4	1-3_strip	1000	0.03998	0.03860	0.04135
ROI6	1-1_strip	859	0.02969	0.02813	0.03125
ROI6	1-2_strip	992	0.02879	0.02789	0.02970
ROI6	1-3_strip	1000	0.03692	0.03550	0.03834
ROI8	1-1_strip	991	0.07627	0.07224	0.08029
ROI8	1-2_strip	930	0.05248	0.04966	0.05531
ROI8	1-3_strip	982	0.03743	0.03574	0.03913

Supplementary Table 3. Descriptive statistics and results of tests for statistical significance for the difference in mean signal intensity in the Lectin reference channel across staining and stripping rounds for the primary validation dataset (**Supplementary Fig. 2d**). Data are from 7 fields of view, 4 of which have were imaged after stripping the first 3 rounds, from one wild-type mouse.

i) Descriptive statistics for the mean signal intensity in the Lectin reference channel.

	**Number of values**	**Mean**	**Std. Deviation**	**Std. Error of Mean**	**Lower 95% CI of mean**	**Upper 95% CI of mean**
**Round 1**	7	196	50.65	19.14	149.1	242.8
**Round 1, Strip + Restain**	4	107	23.92	11.96	68.99	145.1
**Round 2**	7	104.4	28.31	10.7	78.19	130.6
**Round 2, Strip + Restain**	4	47.46	17.5	8.75	19.61	75.3
**Round 3**	7	147	27.89	10.54	121.2	172.8

**Round 3, Strip + Restain**	4	68.98	12.1	6.05	49.72	88.23
**Round 4**	7	72.54	19.71	7.451	54.31	90.77
**Round 5**	7	78.32	20.56	7.77	59.31	97.33
**Round 6**	7	63.93	6.494	2.454	57.92	69.93
**Round 7**	7	66.18	17.01	6.43	50.44	81.91

ii) Summary of mixed effects analysis, performed in GraphPad Prism. Mixed effects analysis was performed instead of one-way ANOVA because stripping rounds were not imaged for some fields of view, leading to missing data.

**Fixed effect (type III)**	**P value**	**P value summary**	**(P < 0.05)?**	**F (DFn, DFd)**
**Treatment (between columns)**	<0.0001	****	Yes	F (1.642, 8.209) = 49.88
**Random effects**	**SD**	**Variance**		
**Individual (between rows)**	19.8	391.9		
**Residual**	16.59	275.3		

iii) Tukey's multiple comparisons test following mixed effects analysis, performed in GraphPad Prism.

**Tukey's multiple comparisons test**	**Mean Diff.**	**95.00% CI of diff.**	**Summary**	**Adjusted P Value**
Round 1 vs. Round 1, Strip	88.91	-47.61 to 225.4	ns	0.1518
Round 1 vs. Round 2	91.58	37.73 to 145.4	**	0.0036
Round 1 vs. Round 2, Strip	148.5	2.307 to 294.7	*	0.0479
Round 1 vs. Round 3	48.92	-0.9132 to 98.76	ns	0.0542
Round 1 vs. Round 3, strip	127.0	-39.93 to 293.9	ns	0.1032
Round 1 vs. Round 4	123.4	67.83 to 179.0	***	0.0008
Round 1 vs. Round 5	117.6	55.57 to 179.7	**	0.0020
Round 1 vs. Round 6	132.0	50.49 to 213.6	**	0.0047
Round 1 vs. Round 7	129.8	66.69 to 192.9	**	0.0013
Round 1, Strip vs. Round 2	2.668	-29.54 to 34.88	ns	0.9993
Round 1, Strip vs. Round 2, Strip	59.59	-8.841 to 128.0	ns	0.0726
Round 1, Strip vs. Round 3	-39.99	-110.6 to 30.64	ns	0.2106
Round 1, Strip vs. Round 3, strip	38.07	-6.565 to 82.70	ns	0.0767
Round 1, Strip vs. Round 4	34.50	-12.88 to 81.88	ns	0.1153
Round 1, Strip vs. Round 5	28.73	-4.903 to 62.36	ns	0.0764
Round 1, Strip vs. Round 6	43.11	-10.57 to 96.80	ns	0.0897
Round 1, Strip vs. Round 7	40.87	6.766 to 74.97	*	0.0303
Round 2 vs. Round 2, Strip	56.92	-12.94 to 126.8	ns	0.0864
Round 2 vs. Round 3	-42.65	-74.01 to -11.30	*	0.0116
Round 2 vs. Round 3, strip	35.40	-26.05 to 96.84	ns	0.2025
Round 2 vs. Round 4	31.83	3.480 to 60.19	*	0.0296

Round 2 vs. Round 5	26.06	-14.07 to 66.18	ns	0.2526
Round 2 vs. Round 6	40.45	-0.05939 to 80.95	ns	0.0503
Round 2 vs. Round 7	38.20	0.5219 to 75.88	*	0.0471
Round 2, Strip vs. Round 3	-99.57	-164.0 to -35.10	*	0.0147
Round 2, Strip vs. Round 3, strip	-21.52	-82.76 to 39.72	ns	0.5213
Round 2, Strip vs. Round 4	-25.09	-66.87 to 16.70	ns	0.1842
Round 2, Strip vs. Round 5	-30.86	-82.70 to 20.98	ns	0.1878
Round 2, Strip vs. Round 6	-16.47	-64.16 to 31.22	ns	0.5347
Round 2, Strip vs. Round 7	-18.72	-59.46 to 22.02	ns	0.3274
Round 3 vs. Round 3, strip	78.05	-17.64 to 173.7	ns	0.0862
Round 3 vs. Round 4	74.49	45.09 to 103.9	***	0.0004
Round 3 vs. Round 5	68.71	30.37 to 107.1	**	0.0027
Round 3 vs. Round 6	83.10	40.11 to 126.1	**	0.0018
Round 3 vs. Round 7	80.85	47.06 to 114.6	***	0.0005
Round 3, strip vs. Round 4	-3.565	-54.68 to 47.55	ns	0.9998
Round 3, strip vs. Round 5	-9.340	-51.43 to 32.75	ns	0.8430
Round 3, strip vs. Round 6	5.049	-14.13 to 24.23	ns	0.7391
Round 3, strip vs. Round 7	2.801	-36.19 to 41.79	ns	0.9997
Round 4 vs. Round 5	-5.775	-23.59 to 12.04	ns	0.8586
Round 4 vs. Round 6	8.613	-19.26 to 36.49	ns	0.8841
Round 4 vs. Round 7	6.366	-8.215 to 20.95	ns	0.6239
Round 5 vs. Round 6	14.39	-15.15 to 43.92	ns	0.5158
Round 5 vs. Round 7	12.14	4.054 to 20.23	**	0.0070
Round 6 vs. Round 7	-2.248	-26.27 to 21.77	ns	>0.9999

Supplementary Table 4. Registration error for second validation dataset, measured using the reference channel (**Supplementary Fig. 3**). Registration error measured using the other channels (SynGAP, NMDAR1) was similar. We note that the first attempt at obtaining round009 failed to produce signal due to experimenter error (likely omission of an antibody); thus, the attempt was repeated and another round of stripping and staining obtained).

**Field of view name**	**Round pair**	**# of ROIs after outlier removal**	**Mean (um)**	**Lower 95% of mean (um)**	**Upper 95% of mean (um)**
ROI1	1-1-strip	990	0.04034	0.0388	0.04188
ROI1	1-2	986	0.02974	0.02889	0.03059
ROI1	1-2-strip	999	0.02973	0.02856	0.03089
ROI1	1-3	977	0.04413	0.04278	0.04549
ROI1	1-3-strip	992	0.03971	0.03839	0.04103
ROI1	1-4	977	0.05041	0.04898	0.05183
ROI1	1-4-strip	988	0.04952	0.04791	0.05112

ROI1	1-5	924	0.07256	0.06927	0.07585
ROI1	1-5-strip	994	0.06316	0.06103	0.06529
ROI1	1-6	932	0.04705	0.04521	0.04889
ROI1	1-6-strip	877	0.06912	0.06595	0.07229
ROI1	1-7	990	0.04464	0.04315	0.04612
ROI1	1-7-strip	999	0.08439	0.08158	0.0872
ROI1	1-8	969	0.04214	0.04083	0.04346
ROI1	1-8-strip	986	0.0493	0.04745	0.05115
ROI1	1-9	946	0.09552	0.09277	0.09827
ROI1	1-9-strip	987	0.0754	0.07346	0.07734
ROI1	1-10	980	0.04567	0.04423	0.0471
ROI2	1-1-strip	915	0.07328	0.07055	0.07601
ROI2	1-2	995	0.04697	0.04548	0.04846
ROI2	1-2-strip	966	0.1236	0.1186	0.1287
ROI2	1-3	988	0.04343	0.04186	0.045
ROI2	1-3-strip	1000	0.2629	0.2489	0.2769
ROI2	1-4	996	0.06795	0.06547	0.07043
ROI2	1-4-strip	948	0.1098	0.106	0.1137
ROI2	1-5	933	0.08648	0.08344	0.08953
ROI2	1-5-strip	913	0.1902	0.1826	0.1978
ROI2	1-6	949	0.08814	0.08433	0.09196
ROI2	1-6-strip	941	0.1225	0.1192	0.1258
ROI2	1-7	809	0.09866	0.09141	0.1059
ROI2	1-7-strip	948	0.1999	0.1928	0.2070
ROI2	1-8	939	0.05672	0.05433	0.05912
ROI2	1-8-strip	949	0.1515	0.1455	0.1575
ROI2	1-9	966	0.07434	0.07161	0.07706

ROI2	1-9-strip	951	0.2236	0.2154	0.2319
ROI2	1-10	970	0.07714	0.074	0.08029
ROI3	1-1-strip	955	0.02038	0.01947	0.0213
ROI3	1-2	997	0.02382	0.02262	0.02503
ROI3	1-2-strip	1000	0.03003	0.02894	0.03111
ROI3	1-3	993	0.06608	0.06422	0.06794
ROI3	1-3-strip	973	0.02478	0.02348	0.02607
ROI3	1-4	934	0.06436	0.06254	0.06618
ROI3	1-4-strip	730	0.04095	0.03841	0.04349
ROI3	1-5	867	0.1445	0.1398	0.1492
ROI3	1-5-strip	992	0.04866	0.04645	0.05086
ROI3	1-6	995	0.05271	0.04948	0.05594
ROI3	1-6-strip	998	0.05627	0.05417	0.05838
ROI3	1-7	955	0.09863	0.09451	0.1027
ROI3	1-7-strip	990	0.06217	0.05979	0.06455
ROI3	1-8	996	0.07545	0.07202	0.07889
ROI3	1-8-strip	931	0.04437	0.04235	0.04639
ROI3	1-9	996	0.0755	0.07191	0.07909
ROI3	1-9-strip	925	0.06714	0.06404	0.07024
ROI3	1-10	940	0.06936	0.06643	0.07229

Supplementary Table 5. Protein and channel information for each round of the multiplexed 5xFAD dataset (n = 2 mice per condition) (**Figs. 3-4**).

	**ch1 (633)**	**ch2 (546)**	**ch3 (488)**
**Round 1**	Lectin/SMI/GFAP	RIM	AB6E10
**Round 2**	Lectin/SMI/GFAP	NR2B	GluA3
**Round 3**	Lectin/SMI/GFAP	RIM-BP	GluA2

**Round 4**	Lectin/SMI/GFAP	GluA1	NR1
**Round 5**	Lectin/SMI/GFAP	PLP1	Shank3
**Round 6**	Lectin/SMI/GFAP	Homer1	CaMKII
**Round 7**	Lectin/SMI/GFAP	D54D2	Cav2.1
**Round 8**	Lectin/SMI/GFAP	GluA4	12F4
**Round 9**	Lectin/SMI/GFAP	PSD95	Bassoon
**Round 10**	Lectin/SMI/GFAP	SynGAP	IRsp53
**Round 11**	Lectin/SMI/GFAP	Stargazin	Gephyrin*

*Excluded due to poor staining quality.

Supplementary Table 6. Registration error for the multiplexed 5xFAD vs. WT mouse dataset (n = 2 mice per condition), measured using the combined reference channel (**Figs. 3-4**).

**Field of view name**	**Round pair**	**# of subvolumes after outlier removal**	**Mean (um)**	**Lower 95% CI (um)**	**Upper 95% CI (um)**
S1ROI1 (5xFAD)	1-2	1000	0.05357	0.05184	0.05529
S1ROI1 (5xFAD)	1-3	1000	0.06229	0.0601	0.06449
S1ROI1 (5xFAD)	1-4	1000	0.088	0.08523	0.09077
S1ROI1 (5xFAD)	1-5	998	0.09537	0.09192	0.09882
S1ROI1 (5xFAD)	1-6	991	0.1052	0.1013	0.1091
S1ROI1 (5xFAD)	1-7	973	0.1047	0.1003	0.1091
S1ROI1 (5xFAD)	1-8	988	0.1507	0.1445	0.1568
S1ROI1 (5xFAD)	1-9	967	0.1477	0.1419	0.1536
S1ROI1 (5xFAD)	1-10	889	0.1251	0.1191	0.1311
S1ROI1 (5xFAD)	1-11	969	0.1503	0.1433	0.1574
S1ROI2 (5xFAD)	1-2	1000	0.02343	0.02258	0.02428
S1ROI2 (5xFAD)	1-3	1000	0.02922	0.02817	0.03026
S1ROI2 (5xFAD)	1-4	1000	0.03536	0.03402	0.03669

S1ROI2 (5xFAD)	1-5	1000	0.0405	0.03892	0.04208
S1ROI2 (5xFAD)	1-6	998	0.03929	0.03752	0.04106
S1ROI2 (5xFAD)	1-7	999	0.04371	0.0418	0.04562
S1ROI2 (5xFAD)	1-8	1000	0.04854	0.04642	0.05065
S1ROI2 (5xFAD)	1-9	1000	0.04845	0.04599	0.05091
S1ROI2 (5xFAD)	1-10	959	0.09445	0.09148	0.09743
S1ROI2 (5xFAD)	1-11	999	0.05537	0.05312	0.05762
S1ROI3 (5xFAD)	1-2	998	0.0138	0.01311	0.01448
S1ROI3 (5xFAD)	1-3	1000	0.01228	0.01166	0.0129
S1ROI3 (5xFAD)	1-4	975	0.02152	0.02072	0.02232
S1ROI3 (5xFAD)	1-5	1000	0.01882	0.01787	0.01976
S1ROI3 (5xFAD)	1-6	991	0.0241	0.02296	0.02525
S1ROI3 (5xFAD)	1-7	760	0.006232	0.00568	0.006783
S1ROI3 (5xFAD)	1-8	994	0.02094	0.01983	0.02204
S1ROI3 (5xFAD)	1-9	982	0.02181	0.02059	0.02304
S1ROI3 (5xFAD)	1-10	839	0.01284	0.01188	0.0138
S1ROI3 (5xFAD)	1-11	994	0.02612	0.02493	0.02731
S1ROI4 (5xFAD)	1-2	998	0.03358	0.03166	0.03549
S1ROI4 (5xFAD)	1-3	815	0.02288	0.02151	0.02426
S1ROI4 (5xFAD)	1-4	995	0.06163	0.05836	0.0649
S1ROI4 (5xFAD)	1-5	749	0.02593	0.02477	0.02708
S1ROI4 (5xFAD)	1-6	761	0.02315	0.022	0.0243
S1ROI4 (5xFAD)	1-7	787	0.02676	0.02544	0.02807
S1ROI4 (5xFAD)	1-8	777	0.02663	0.02533	0.02792
S1ROI4 (5xFAD)	1-9	778	0.03155	0.03029	0.03281
S1ROI4 (5xFAD)	1-10	768	0.03991	0.03767	0.04215
S1ROI4 (5xFAD)	1-11	797	0.0409	0.03895	0.04286

S1ROI5 (5xFAD)	1-2	1000	0.04371	0.04268	0.04475
S1ROI5 (5xFAD)	1-3	1000	0.04652	0.04512	0.04792
S1ROI5 (5xFAD)	1-4	1000	0.06155	0.05983	0.06326
S1ROI5 (5xFAD)	1-5	1000	0.06824	0.06608	0.0704
S1ROI5 (5xFAD)	1-6	1000	0.07194	0.06977	0.0741
S1ROI5 (5xFAD)	1-7	998	0.0833	0.0809	0.08569
S1ROI5 (5xFAD)	1-8	999	0.08965	0.08635	0.09295
S1ROI5 (5xFAD)	1-9	991	0.1011	0.09778	0.1045
S1ROI5 (5xFAD)	1-10	988	0.09982	0.09654	0.1031
S1ROI5 (5xFAD)	1-11	995	0.1024	0.09895	0.1058
S2ROI1 (5xFAD)	1-2	0.0233	0.02257	0.02404	1000
S2ROI1 (5xFAD)	1-3	0.02564	0.02473	0.02656	1000
S2ROI1 (5xFAD)	1-4	0.03254	0.03133	0.03375	1000
S2ROI1 (5xFAD)	1-5	0.03394	0.03262	0.03525	1000
S2ROI1 (5xFAD)	1-6	0.03902	0.0376	0.04045	1000
S2ROI1 (5xFAD)	1-7	0.04912	0.04729	0.05095	1000
S2ROI1 (5xFAD)	1-8	0.04632	0.04456	0.04807	1000
S2ROI1 (5xFAD)	1-9	0.05088	0.04894	0.05281	1000
S2ROI1 (5xFAD)	1-10	0.05191	0.04976	0.05407	1000
S2ROI1 (5xFAD)	1-11	0.06204	0.05985	0.06422	1000
S2ROI2 (5xFAD)	1-2	0.01522	0.0147	0.01573	913
S2ROI2 (5xFAD)	1-3	0.02099	0.01989	0.02208	1000
S2ROI2 (5xFAD)	1-4	0.02614	0.02515	0.02713	939
S2ROI2 (5xFAD)	1-5	0.02547	0.02438	0.02655	938
S2ROI2 (5xFAD)	1-6	0.03137	0.02994	0.0328	934
S2ROI2 (5xFAD)	1-7	0.02583	0.02433	0.02734	921
S2ROI2 (5xFAD)	1-8	0.04117	0.03896	0.04338	977

S2ROI2 (5xFAD)	1-9	0.03847	0.03647	0.04047	971
S2ROI2 (5xFAD)	1-10	0.03335	0.03144	0.03526	915
S2ROI2 (5xFAD)	1-11	0.04357	0.04153	0.04561	951
S2ROI3 (5xFAD)	1-2	0.01803	0.01722	0.01883	977
S2ROI3 (5xFAD)	1-3	0.02098	0.02013	0.02183	923
S2ROI3 (5xFAD)	1-4	0.02133	0.02041	0.02225	917
S2ROI3 (5xFAD)	1-5	0.02656	0.02537	0.02775	992
S2ROI3 (5xFAD)	1-6	0.02985	0.02863	0.03107	991
S2ROI3 (5xFAD)	1-7	0.02725	0.02599	0.02852	898
S2ROI3 (5xFAD)	1-8	0.02217	0.02133	0.023	883
S2ROI3 (5xFAD)	1-9	0.03627	0.03458	0.03796	942
S2ROI3 (5xFAD)	1-10	0.03572	0.03418	0.03725	934
S2ROI3 (5xFAD)	1-11	0.03977	0.0378	0.04174	954
S2ROI4 (5xFAD)	1-2	0.03583	0.03469	0.03698	979
S2ROI4 (5xFAD)	1-3	0.04729	0.04588	0.0487	1000
S2ROI4 (5xFAD)	1-4	0.0696	0.06738	0.07183	981
S2ROI4 (5xFAD)	1-5	0.07327	0.07153	0.07501	928
S2ROI4 (5xFAD)	1-6	0.08729	0.08485	0.08972	992
S2ROI4 (5xFAD)	1-7	0.1002	0.09772	0.1027	997
S2ROI4 (5xFAD)	1-8	0.1175	0.114	0.121	991
S2ROI4 (5xFAD)	1-9	0.1238	0.1202	0.1273	944
S2ROI4 (5xFAD)	1-10	0.1023	0.1001	0.1045	914
S2ROI4 (5xFAD)	1-11	0.1292	0.1262	0.1323	998
S3ROI1 (WT)	1-2	0.04098	0.03906	0.04289	998
S3ROI1 (WT)	1-3	0.02568	0.02481	0.02656	995
S3ROI1 (WT)	1-4	0.01959	0.01892	0.02027	972
S3ROI1 (WT)	1-5	0.02696	0.02616	0.02776	995

S3ROI1 (WT)	1-6	0.05027	0.04846	0.05207	1000
S3ROI1 (WT)	1-7	0.05638	0.05437	0.05839	1000
S3ROI1 (WT)	1-8	0.04515	0.04402	0.04627	996
S3ROI1 (WT)	1-9	0.04669	0.04527	0.04811	999
S3ROI1 (WT)	1-10	0.05092	0.04931	0.05253	1000
S3ROI1 (WT)	1-11	0.0459	0.04474	0.04705	998
S3ROI2 (WT)	1-2	0.01304	0.01264	0.01344	990
S3ROI2 (WT)	1-3	0.01944	0.01881	0.02007	1000
S3ROI2 (WT)	1-4	0.02082	0.02033	0.02131	1000
S3ROI2 (WT)	1-5	0.01391	0.01345	0.01437	951
S3ROI2 (WT)	1-6	0.02173	0.02107	0.02238	999
S3ROI2 (WT)	1-7	0.03299	0.03202	0.03397	993
S3ROI2 (WT)	1-8	0.02239	0.02163	0.02315	1000
S3ROI2 (WT)	1-9	0.01558	0.01505	0.01611	879
S3ROI2 (WT)	1-10	0.03076	0.02979	0.03172	1000
S3ROI2 (WT)	1-11	0.01898	0.01835	0.01961	995
S3ROI3 (WT)	1-2	0.01005	0.009553	0.01054	1000
S3ROI3 (WT)	1-3	0.0161	0.01559	0.0166	996
S3ROI3 (WT)	1-4	0.01443	0.01382	0.01503	1000
S3ROI3 (WT)	1-5	0.02465	0.02398	0.02532	995
S3ROI3 (WT)	1-6	0.01783	0.01707	0.01858	1000
S3ROI3 (WT)	1-7	0.0291	0.02826	0.02993	981
S3ROI3 (WT)	1-8	0.03132	0.03026	0.03238	1000
S3ROI3 (WT)	1-9	0.02515	0.02432	0.02599	979
S3ROI3 (WT)	1-10	0.02341	0.02252	0.0243	967
S3ROI3 (WT)	1-11	0.03025	0.02941	0.03109	984
S3ROI4 (WT)	1-2	0.01448	0.01405	0.01491	979

S3ROI4 (WT)	1-3	0.01397	0.01331	0.01462	1000
S3ROI4 (WT)	1-4	0.02117	0.02035	0.02199	972
S3ROI4 (WT)	1-5	0.01439	0.01378	0.01501	975
S3ROI4 (WT)	1-6	0.0187	0.01791	0.01949	990
S3ROI4 (WT)	1-7	0.01555	0.01503	0.01608	847
S3ROI4 (WT)	1-8	0.01877	0.01788	0.01965	972
S3ROI4 (WT)	1-9	0.01853	0.01778	0.01928	973
S3ROI4 (WT)	1-10	0.02762	0.02669	0.02855	949
S3ROI4 (WT)	1-11	0.01988	0.01908	0.02068	951
S4ROI1 (WT)	1-2	0.02937	0.02862	0.03013	994
S4ROI1 (WT)	1-3	0.04884	0.0473	0.05038	976
S4ROI1 (WT)	1-4	0.04548	0.04393	0.04703	1000
S4ROI1 (WT)	1-5	0.0443	0.04287	0.04572	1000
S4ROI1 (WT)	1-6	0.04846	0.04697	0.04995	1000
S4ROI1 (WT)	1-7	0.05113	0.04914	0.05311	1000
S4ROI1 (WT)	1-8	0.06986	0.06773	0.07198	1000
S4ROI1 (WT)	1-9	0.06512	0.06304	0.06721	997
S4ROI1 (WT)	1-10	0.07089	0.06835	0.07343	982
S4ROI1 (WT)	1-11	0.07369	0.07111	0.07628	996
S4ROI2 (WT)	1-2	0.02356	0.02282	0.02431	1000
S4ROI2 (WT)	1-3	0.03513	0.03394	0.03632	999
S4ROI2 (WT)	1-4	0.03518	0.03382	0.03653	998
S4ROI2 (WT)	1-5	0.04005	0.03866	0.04145	999
S4ROI2 (WT)	1-6	0.04043	0.039	0.04186	998
S4ROI2 (WT)	1-7	0.05032	0.04826	0.05238	993
S4ROI2 (WT)	1-8	0.0526	0.05048	0.05471	999
S4ROI2 (WT)	1-9	0.05509	0.05285	0.05732	995

S4ROI2 (WT)	1-10	0.06117	0.0585	0.06383	995
S4ROI2 (WT)	1-11	0.05886	0.0564	0.06133	998
S4ROI3 (WT)	1-2	0.03682	0.03558	0.03807	1000
S4ROI3 (WT)	1-3	0.04038	0.03908	0.04168	1000
S4ROI3 (WT)	1-4	0.04308	0.04154	0.04462	995
S4ROI3 (WT)	1-5	0.04911	0.04741	0.05082	1000
S4ROI3 (WT)	1-6	0.04884	0.04671	0.05096	994
S4ROI3 (WT)	1-7	0.05744	0.05534	0.05954	995
S4ROI3 (WT)	1-8	0.07016	0.06796	0.07235	994
S4ROI3 (WT)	1-9	0.07449	0.07166	0.07732	996
S4ROI3 (WT)	1-10	0.07017	0.06755	0.0728	990
S4ROI3 (WT)	1-11	0.07374	0.07071	0.07678	972
S4ROI4 (WT)	1-2	0.009341	0.008858	0.009825	999
S4ROI4 (WT)	1-3	0.0107	0.01005	0.01135	994
S4ROI4 (WT)	1-4	0.01402	0.01325	0.01479	1000
S4ROI4 (WT)	1-5	0.01694	0.01613	0.01775	1000
S4ROI4 (WT)	1-6	0.01674	0.01603	0.01745	996
S4ROI4 (WT)	1-7	0.01232	0.0115	0.01313	968
S4ROI4 (WT)	1-8	0.02091	0.01986	0.02196	999
S4ROI4 (WT)	1-9	0.02	0.01898	0.02101	989
S4ROI4 (WT)	1-10	0.02116	0.0201	0.02222	993
S4ROI4 (WT)	1-11	0.02132	0.02029	0.02235	999

Supplementary Table 7. Results of linear mixed effect model, implement in Python's statsmodels package[1], testing the effect of group (5xFAD vs. WT) on total volume of each synaptic protein, with group assignment equal to animal (n = 2 animals per group, n = 4-5 fields of view per animal).

**Protein**	**Group coefficient (WT)**	**Group z statistic**	**Group p-value**
RIM1	0.213	0.841	0.401

NR2B	0.121	4.801	1.58×10^{-6}
GluA3	0.161	4.705	2.54×10^{-6}
RIM-BP	0.199	2.245	0.025
GluA2	-0.029	-3.622	2.92×10^{-4}
GluA1	0.127	4.713	2.439×10^{-8}
NR1	0.142	4.836	1.327×10^{-6}
Shank3	0.248	3.412	6.438×10^{-4}
Homer1	0.124	1.865	0.062
CaMKIIa	-0.057	0.5885	-0.541
Cav2.1	0.155	0.00195	3.098
GluA4	-0.052	-1.438	0.1504
PSD95	0.321	3.441	5.803×10^{-4}
Bassoon	0.307	5.431	5.615×10^{-8}
SynGAP	0.336	7.699	1.372×10^{-14}
IRsp53	0.097	1.851	0.0642
Stargazin	0.096	0.1730	1.363

Supplementary Table 8. Descriptive statistics of Aβ, myelin, and synaptic protein volume within manually-identified Aβ nanocluster ROIs (**Fig. 4b**). Data are from 71 nanocluster ROIs from 9 fields of view from 2 12-month 5xFAD mice.

**Protein**	**Mean volume (um^3)**	**Lower 95% CI (um^3)**	**Upper 95% CI (um^3)**
6E10	0.0002773	0.0002427	0.0003118
D54D2	0.0002918	0.000257	0.0003266
12F4	0.000258	0.000224	0.0002921
RIM1	7.82E-05	5.55E-05	0.000101
NR2B	1.80E-05	1.07E-05	2.53E-05
GluA3	5.89E-05	3.79E-05	7.98E-05
RIM-BP	0.0001055	8.82E-05	0.0001229
GluA2	0.0002211	0.0001851	0.0002571
GluA1	2.35E-05	1.12E-05	3.59E-05

NR1	1.93E-05	5.67E-06	3.30E-05
Shank3	7.57E-05	5.66E-05	9.49E-05
Homer1	0.0001175	9.16E-05	0.0001434
CaMKIIa	0.0001625	0.0001347	0.0001903
Cav2.1	5.99E-05	4.33E-05	7.66E-05
GluA4	6.62E-05	4.02E-05	9.21E-05
PSD95	6.32E-05	4.16E-05	8.48E-05
Bassoon	7.78E-05	5.93E-05	9.64E-05
SynGAP	8.87E-05	6.35E-05	0.0001138
IRSp53	8.44E-05	6.14E-05	0.0001074
Stargazin	3.12E-05	1.40E-05	4.84E-05
PLP1	8.91E-05	6.56E-05	0.0001126

Supplementary Table 9. Statistics accompanying the analysis of GluA1-4 colocalization with D54D2 within manually-identified Aβ nanocluster ROIs (**Fig. 4c-e**). Analysis was conducted in GraphPad Prism.

i) Descriptive statistics of the fraction of volume mutually overlapped with D54D2 (e.g., GluA2 volume overlapped with D54D2 divided by total D54D2 volume, **Fig. 4c**) for all AMPAR subunits. Only ROIs with nonzero volumes of each protein after size filtration are included. For this analysis, we excluded ROIs with visible offset between the Aβ channels, representing residual registration error.

	**GluA2**	**GluA4**	**GluA3**	**GluA1**
Number of values	44	44	44	44
Mean	0.4835	0.1460	0.04257	0.000
Std. Deviation	0.1983	0.1828	0.09443	0.000
Std. Error of Mean	0.02990	0.02756	0.01424	0.000
Lower 95% CI of mean	0.4232	0.09042	0.01386	0.000
Upper 95% CI of mean	0.5438	0.2016	0.07128	0.000

ii) Results of one-way ANOVA followed by Tukey's multiple comparisons test on the fraction of volume mutually overlapped with D54D2 for all AMPAR subunits. Only ROIs with nonzero volumes of each protein after size filtration are included. For this analysis, we excluded ROIs with visible offset between the Aβ channels, representing residual registration error.

**ANOVA table**	**SS**	**DF**	**MS**	**F (DFn, DFd)**	**P value**
Treatment (between columns)	6.336	3	2.112	F(3, 172) = 103.4	P<0.0001

Residual (within columns)	3.512	172	0.02042		
Total	9.848	175			

**Tukey's multiple comparisons test**	**Mean Diff.**	**95.00% CI of diff.**	**Below threshold?**	**Summary**	**Adjusted P Value**
GluA1 vs. GluA2	0.3376	0.2585 to 0.4166	Yes	****	<0.0001
GluA1 vs. GluA3	0.4410	0.3619 to 0.5200	Yes	****	<0.0001
GluA1 vs. GluA4	0.4835	0.4045 to 0.5626	Yes	****	<0.0001
GluA2 vs. GluA3	0.1034	0.02439 to 0.1825	Yes	**	0.0047
GluA2 vs. GluA4	0.1460	0.06696 to 0.2250	Yes	****	<0.0001
GluA3 vs. GluA4	0.04257	-0.03646 to 0.1216	No	ns	0.5027

iii) Results of simple linear regression on the volume of GluA4 or GluA2 vs. D54D2 (**Fig. 4d**).

	**GluA2**	**GluA4**
Number of Values	71	71
Slope, 95% CI	[0.8456,1.046]	[0.3878,0.6465]
R squared	0.8370	0.4796
F	354.3	63.59
P value	<0.0001	<0.0001
Deviation from zero?	Significant	Significant

iv) Results of simple linear regression on the volume of GluA2 vs. GluA4 (**Fig. 4e**).

**95% CI of slope**	[0.8787, 1.292]
**R^2**	0.6143
**P value**	<0.0001
**# of values**	71

Supplementary Table 10. Protein and channel information for each round of multiplexed synaptic dataset (**Fig. 5)**, mice #1 (S1) and #2 (S2).

	**ch1 (633)**	**ch2 (546)**	**ch3 (488)**
Round 1	Lectin/SMI/GFAP	SynGAP	NR1
Round 2	Lectin/SMI/GFAP	RIM1	Gephyrin
Round 3	Lectin/SMI/GFAP	GluA4	IRSp53

Round 4	Lectin/SMI/GFAP	GluA1	NR2B
Round 5	Lectin/SMI/GFAP	Homer1	CaMKIIa
Round 6	Lectin/SMI/GFAP	*no signal	Shank3
Round 7	Lectin/SMI/GFAP	GluA3	Bassoon
Round 8	Lectin/SMI/GFAP	Erbb4	Stargazin
Round 9	Lectin/SMI/GFAP	Elfn1	no signal*
Round 10	Lectin/SMI/GFAP	PSD95	Cav2.1
Round 11	Lectin/SMI/GFAP	GluA2	Vglut1

*Excluded from analysis and visualization due to poor staining quality or were already imaged in a prior round.

Supplementary Table 11. Registration error for multiplexed synaptic dataset, measured using the Lectin/SMI/Homer reference channel (**Fig. 5**). S1: mouse #1, S2: mouse #2.

**Field of view name**	**Round pair**	**# of subvolumes after outlier removal**	**Mean (µm)**	**Lower 95% CI (µm)**	**Upper 95% CI (µm)**
S1ROI1	1-2	1000	0.04259	0.04105	0.04413
S1ROI1	1-3	1000	0.06999	0.06749	0.07248
S1ROI1	1-4	1000	0.0664	0.06432	0.06848
S1ROI1	1-5	1000	0.08332	0.08031	0.08633
S1ROI1	1-6	996	0.07632	0.07346	0.07918
S1ROI1	1-7	999	0.08812	0.08489	0.09135
S1ROI1	1-8	998	0.084	0.08113	0.08688
S1ROI1	1-9	996	0.07642	0.07353	0.0793
S1ROI1	1-10	999	0.08479	0.0814	0.08817
S1ROI1	1-11	994	0.0924	0.08927	0.09554
S1ROI1	1-12	996	0.09051	0.08743	0.0936

S1ROI2	1-2	998	0.04356	0.0424	0.04472
S1ROI2	1-3	1000	0.06717	0.06543	0.06892
S1ROI2	1-4	996	0.06591	0.06407	0.06776
S1ROI2	1-5	997	0.08317	0.08075	0.08559
S1ROI2	1-6	988	0.08298	0.08062	0.08535
S1ROI2	1-7	999	0.08392	0.08144	0.0864
S1ROI2	1-8	987	0.08941	0.08687	0.09194
S1ROI2	1-9	995	0.07788	0.07538	0.08039
S1ROI2	1-10	982	0.1138	0.1096	0.1179
S1ROI2	1-11	987	0.09434	0.09159	0.09709
S1ROI2	1-12	990	0.09294	0.09003	0.09586
S1ROI3	1-2	995	0.02593	0.0247	0.02716
S1ROI3	1-3	985	0.03334	0.03199	0.0347
S1ROI3	1-4	831	0.01928	0.01818	0.02038
S1ROI3	1-5	855	0.02579	0.02457	0.027
S1ROI3	1-6	881	0.0256	0.02423	0.02697
S1ROI3	1-7	974	0.04145	0.03988	0.04303
S1ROI3	1-8	980	0.05178	0.0498	0.05375
S1ROI3	1-9	963	0.05851	0.05692	0.06009
S1ROI3	1-10	968	0.06061	0.05873	0.06249
S1ROI3	1-11	988	0.06048	0.05875	0.0622
S1ROI3	1-12	961	0.0557	0.05388	0.05751
S1ROI4	1-2	998	0.02598	0.02475	0.02721
S1ROI4	1-3	988	0.03342	0.03206	0.03477
S1ROI4	1-4	834	0.01932	0.01821	0.02042
S1ROI4	1-5	858	0.02576	0.02455	0.02697
S1ROI4	1-6	883	0.02561	0.02424	0.02698

S1ROI4	1-7	976	0.04145	0.03988	0.04302
S1ROI4	1-8	982	0.05173	0.04976	0.05371
S1ROI4	1-9	964	0.05854	0.05697	0.06012
S1ROI4	1-10	971	0.06054	0.05867	0.06242
S1ROI4	1-11	991	0.06048	0.05876	0.0622
S1ROI4	1-12	964	0.05569	0.05388	0.0575
S2ROI1	1-2	944	0.0217	0.02087	0.02253
S2ROI1	1-3	996	0.03027	0.02878	0.03175
S2ROI1	1-4	983	0.04392	0.04222	0.04561
S2ROI1	1-5	898	0.02229	0.02118	0.02339
S2ROI1	1-6	863	0.02165	0.02059	0.0227
S2ROI1	1-7	914	0.04288	0.04097	0.04478
S2ROI1	1-8	991	0.05661	0.05446	0.05876
S2ROI1	1-9	858	0.03626	0.03443	0.0381
S2ROI1	1-10	984	0.04589	0.04355	0.04824
S2ROI1	1-11	848	0.02941	0.02806	0.03075
S2ROI1	1-12	985	0.0494	0.04728	0.05151
S2ROI2	1-2	944	0.0217	0.02087	0.02253
S2ROI2	1-3	996	0.03027	0.02878	0.03175
S2ROI2	1-4	983	0.04392	0.04222	0.04561
S2ROI2	1-5	898	0.02229	0.02118	0.02339
S2ROI2	1-6	863	0.02165	0.02059	0.0227
S2ROI2	1-7	914	0.04288	0.04097	0.04478
S2ROI2	1-8	991	0.05661	0.05446	0.05876
S2ROI2	1-9	858	0.03626	0.03443	0.0381
S2ROI2	1-10	984	0.04589	0.04355	0.04824
S2ROI2	1-11	848	0.02941	0.02806	0.03075

S2ROI2	1-12	985	0.0494	0.04728	0.05151
S2ROI3	1-2	1000	0.0452	0.04356	0.04684
S2ROI3	1-3	999	0.04059	0.03893	0.04224
S2ROI3	1-4	1000	0.05878	0.05684	0.06072
S2ROI3	1-5	1000	0.05668	0.05485	0.05851
S2ROI3	1-6	998	0.04476	0.04285	0.04667
S2ROI3	1-7	983	0.05921	0.0567	0.06173
S2ROI3	1-8	994	0.0619	0.05964	0.06416
S2ROI3	1-9	941	0.04499	0.04305	0.04692
S2ROI3	1-10	987	0.0791	0.07618	0.08202
S2ROI3	1-11	987	0.05464	0.05243	0.05686
S2ROI3	1-12	998	0.05848	0.05622	0.06075
S2ROI4	1-2	981	0.02405	0.02309	0.02502
S2ROI4	1-3	999	0.02246	0.02165	0.02328
S2ROI4	1-4	961	0.0238	0.02294	0.02465
S2ROI4	1-5	995	0.02518	0.02396	0.0264
S2ROI4	1-6	971	0.02133	0.02056	0.0221
S2ROI4	1-7	916	0.02806	0.0271	0.02903
S2ROI4	1-8	960	0.0242	0.02319	0.02521
S2ROI4	1-9	961	0.0251	0.02438	0.02582
S2ROI4	1-10	939	0.03724	0.03555	0.03892
S2ROI4	1-11	987	0.02417	0.02301	0.02533
S2ROI4	1-12	974	0.02869	0.02755	0.02984

Supplementary Table 12. Protein and channel information for each round of multiplexed cultured neuron dataset (**Supplementary Fig. 4**).

	**ch1 (633)**	**ch2 (546)**	**ch3 (488)**

**Round 1**	SMI/GFAP/Homer/Lectin*	Synapsin 1	NR1
**Round 2**	SMI/GFAP/Homer	NR2B	SynGAP
**Round 3**	SMI/GFAP/Homer	GluA1	PSD95
**Round 4**	SMI/GFAP/Homer	Bassoon	Gephyrin
**Round 5**	SMI/GFAP/Homer	RIM1	CaMKIIa

*Lectin was excluded from the reference channel in later rounds after negligible immunoreactivity was confirmed, as neuronal cultures are not vascularized.

Supplementary Table 13. Registration error for multiplexed cultured neuron dataset, measured using the SMI/GFAP/Homer reference channel (**Supplementary Fig. 4**).

**Field of view name**	**Round pair**	**# of subvolumes after outlier removal**	**Mean (μm)**	**Lower 95% CI (μm)**	**Upper 95% CI (μm)**
ROI1	1-2	1000	0.05823	0.05599	0.06047
ROI1	1-3	997	0.05513	0.05297	0.05728
ROI1	1-4	997	0.05094	0.04908	0.0528
ROI1	1-5	934	0.05512	0.05303	0.05721
ROI2	1-2	921	0.03495	0.03392	0.03598
ROI2	1-3	938	0.04504	0.04364	0.04645
ROI2	1-4	961	0.04983	0.04809	0.05156
ROI2	1-5	964	0.03435	0.03304	0.03566
ROI3	1-2	936	0.03399	0.03246	0.03552
ROI3	1-3	999	0.04548	0.04357	0.04739
ROI3	1-4	1000	0.03797	0.03619	0.03975
ROI3	1-5	1000	0.0275	0.02643	0.02858
ROI4	1-2	1000	0.04739	0.04604	0.04874
ROI4	1-3	965	0.05584	0.05409	0.0576

ROI4	1-4	998	0.0594	0.05751	0.06129
ROI4	1-5	1000	0.05947	0.05762	0.06132

Supplementary Table 14. Antibodies that failed with multiExR.

Primary / Secondary	Target	Host	Vendor	Product number	Dilution Factor
Primary	mGluR5	Chicken	Aveslabs	ER5	1:200
Primary	Adam22	Mouse	Antibodies inc	75-093	1:200
Primary	GABA-B	Guinea pig	Millipore Sigma	AB2256	1:200
Primary	CACNA1G	Rabbit	Fisher Scientific	50-173-1816	1:200
Primary	CACNG8	Rabbit	Alamone labs	ACC-125	1:200

Supplementary Table 15. Absolute intensity thresholds used to create binary image volumes for Aβ abundance quantification (**Fig. 3**, see Methods).

Protein	Intensity threshold
D54D2	826
12F4	288
6E10	639

Supplementary Table 16. Absolute intensity thresholds used to create binary image volumes for synaptic protein abundance quantification (**Fig. 3**).

Protein	Intensity threshold
RIM1	259

GluA3	77
NR2B	94
RIM-BP	225
GluA2	40
GluA1	75
NR1	61
Shank3	188
Homer1	479
CaMKIIa	486
Cav2.1	111
GluA4	55
PSD95	379
Bassoon	254
SynGAP	1288
IRSp53	153
Stargazin	71

Supplementary Table 17. List of chemicals

Product Name	Vendor	Product Number
Sodium acrylate	Santa Cruz	CAS7446-81-3
Acrylamide	Sigma	A9099
N,N′-Methylenebisacrylamide (BIS)	Sigma	M7279
Ammonium persulfate (APS)	Sigma	A3678

N,N,N′,N′-Tetramethylethylenediamine (TEMED)	Sigma	T7024
4-Hydroxy-TEMPO (HT)	Sigma	176141
6-((acryloyl)amino)hexanoic Acid, Succinimidyl Ester (AcX)	Thermo Fisher	A20770
Sodium dodecyl sulfate (SDS)	Sigma	436143
Sodium Chloride (NaCl)	Thermo Fisher	AM9760
Tris Buffer, pH 8.0	Fisher scientific	77-86-1
Paraformaldehyde	Electron Microscopy Sciences	15710
Triton X-100	Sigma	X100
Glycine	Sigma	50046
PBS 10x	Thermo Fisher	70011044
Normal Donkey Serum	Jackson ImmunoResearch	017-000-121
Sodium citrate dihydrate	Sigma	W302600

Supplementary Table 18. List of antibodies

Primary / Secondary	Target	Host	Vendor	Product number	Dilution	Validation
Primary	Cav1.2	Guinea pig	Synaptic Systems	152 205	0.18055556	P[13], VI

Primary	RIM1	Rabbit	Synaptic Systems	140 003	0.18055556	P[44], VI, KD
Primary	PSD95	Mouse	Thermo Fisher	MA1-046	0.18055556	P[230], VI, KO
Primary	PSD95	Rabbit	Cell Signaling Technology	CST3450S	0.18055556	P[295], VI, R
Primary	SynGAP	Rabbit	Thermo Fisher	PA1-046	0.18055556	P[31], VI, KO
Primary	Homer1	Rabbit	Synaptic Systems	160 003	0.18055556	P[141], VI, KO
Primary	Homer1	Chicken	Synaptic Systems	160 006	0.18055556	P[22], VI, KO
Primary	Bassoon	Guinea pig	Synaptic Systems	141 004	0.18055556	No longer in stock*
Primary	Shank3	Guinea pig	Synaptic Systems	162 304	0.18055556	P[22], VI, KO
Primary	Gephyrin	Mouse	Synaptic Systems	147 011	0.18055556	P[206], VI, KO
Primary	GFAP	Chicken	Abcam	ab4674	0.18055556	P[522], VI
Primary	GluA1	Rabbit	Abcam	ab31232	0.18055556	P[143], VI, KO, R
Primary	CaMKII	Mouse	Abcam	ab22609	0.18055556	P[78], VI
Primary	Synapsin1	Rabbit	Abcam	ab8	0.18055556	P[52], VI, R
Primary	NMDAR1	Mouse	ThermoFisher	32-050-0	0.18055556	P[48], VI
Primary	VGlut	Rabbit	Synaptic Systems	131 011	0.18055556	P[187], VI, KO

Primary	NR2B	Mouse	antibodiesinc	75-101	0.18055556	P[94], VI, KO
Primary	GluA4	Rabbit	Cell Signaling Technology	#8070S	0.18055556	P[15], VI, R
Primary	GluA2	Mouse	antibodiesinc	75-002	0.18055556	P[189], VI, KO
Primary	PLP	Rabbit	Abcam	ab28486	0.18055556	P[78], VI
Primary	Stargazin	Mouse	ThermoFisher	PIMA527645	0.18055556	VI
Primary	Stargazin	Rabbit	Cell Signaling Technology	#8511	0.18055556	P[5], VI, R
Primary	GluA3	Mouse	ThermoFisher	32-040-0	0.18055556	P[8], VI, KO
Primary	GluA3	Rabbit	Abcam	ab40845	0.18055556	P[19], VI
Primary	RIM-BP	Rabbit	Synaptic Systems	316 103	0.18055556	P[9], VI, KO
Primary	Aβ42 (6E10)	Mouse	BioLegend	SIG39320	0.18055556	P[331], VI
Primary	Aβ42 (12F4)	Mouse	BioLegend	SIG39142	0.18055556	P[31], VI
Primary	Aβ42 (D54D2)	Rabbit	Cell Signaling Technology	CST8243S	0.18055556	P[116], VI, R
Primary	SMI	Chicken	Abcam	ab4680	0.31944444	P[118], VI, R
Primary	SMI	Chicken	BioLegend	822601	0.18055556	P[9], VI
Primary	Kv7.2	Mouse	Santa Cruz	sc-271852	0.18055556	P[7], VI, R
Primary	Nav1.6	Rabbit	Abcam	ab65166	0.18055556	P[9], VI, R
Primary	Erbb4	Rabbit	Cell Signaling Technology	CST4795	0.18055556	P[81], VI
Primary	Elfn1	Rabbit	Synaptic Systems	448 003	0.18055556	P[2], VI, KO

Secondary	Mouse	Goat	ThermoFisher	A28175 (Alexa Fluor 488 nm)	1:200	N/A
Secondary	Mouse	Goat	ThermoFisher	A11031 (Alexa Fluor 546 nm)	1:200	N/A
Secondary	Mouse	Donkey	Biotium	20124 (CF 633 nm)	1:200	N/A
Secondary	Mouse	Donkey	ThermoFisher	A10036 (Alexa Fluor 546 nm)	1:200	N/A
Secondary	Rabbit	Goat	ThermoFisher	A11034 (Alexa Fluor 488 nm)	1:200	N/A
Secondary	Rabbit	Goat	ThermoFisher	A11035 (Alexa Fluor 546 nm)	1:200	N/A
Secondary	Rabbit	Donkey	Biotium	20125 (CF 633 nm)	1:200	N/A
Secondary	Rabbit	Donkey	ThermoFisher	A10040 (Alexa Fluor 546 nm)	1:200	N/A
Secondary	Guinea pig	Donkey	Biotium	20171 (CF 633 nm)	1:200	N/A
Secondary	Chicken	Goat	ThermoFisher	A11039 (Alexa Fluor 488 nm)	1:200	N/A
Secondary	Chicken	Donkey	Biotium	20168 (CF 633 nm)	1:200	N/A

P: publications, number of references in superscript, VI: vendor image(s), KO: knock-out, KD: knock-down, R: high user rating (4 or more stars out of 5).
*Its replacement, Synaptic Systems 141 318 is supported by KO, P, and VI.

Supplementary Table 19. Gel solution of ExR

Monomer solution:

Component	Stock Concentration*	Amount (mL)
Sodium acrylate	33% (w/w)*	9
Acrylamide	50% (w/w)*	2
Sodium Chloride	5 M	16
PBS	10x	4
Water	-	3.6
Total	-	34.6

*As weight/weight. For instance, 10 g sodium acrylate powder was dissolved in 20 mL deionized water as 33% (w/w) stock solution. 10 g acrylamide powder was dissolved in 10 mL deionized water as 50% (w/w) stock solution.

Gelling solution:

Reagent	Stock Concentration	1st gel solution (µL)	Re-embedding solution (µL)	3rd gel solution (µL)	Additional re-embedding solution (µL)
Monomer	-	864	-	864	-
Acrylamide	50% w/w	-	275	-	40
Bis acrylamide	1.96% w/w	40	18.75	20	18.75
Water	-	36	701.25	111	936.25
4HT	0.50% w/w	20	-	-	-
TEMED	8% w/w	20	2.5	2.5	2.5
APS	9.10% w/w	20	2.5	2.5	2.5
Total (mL)	-	1	1	1	1

Denaturation buffer:

Reagent	Stock Concentration	Amount (mL)

Sodium dodecyl sulfate (SDS)	700 mM	11.43
Water	-	24.97
Tris buffer, pH 8.0	1 M	2
Sodium Chloride	5 M	1.6
Total	-	40

Supplementary Note 1

We take the mean of all pixels in the image weighted by their similarity to the target pixel. Consider an image having an area Ω and points (p, q) in the image. The filtered values at a point p is

$$u(p) = \frac{1}{C(p)} \int_{\Omega} v(q) f(p, q) dq \quad \forall\, q \in \Omega$$

Here $f(p, q)$ is the weighting function of choice and $C(p)$ is the normalization factor.

$$C(p) = \int_{\Omega} f(p, q)\, dq$$

In our case, we choose $f(p, q)$ to be a Gaussian weighting function

$$f(p, q) = e^{-\frac{|B(q) - B(p)|^2}{h^2}}$$

Whose standard deviation is h which serves as the filtering parameter, and $B(\cdot)$ is the local mean of an image point.

Supplementary Note 2

Since images obtained from expansion sequencing have inhomogenous lighting, we propose to make use of local (or adaptive thresholding) where we define a threshold T for neighboring regions of a pixel at location (i, j).

Within this neighborhood, we replace pixels with a binary mask subject to intensity $I_{i,j}$subject to the following piecewise condition:

$$f(i, j) = 0 \qquad T > I_{i,j}$$

$$255 \quad T < I_{i,j}$$

Supplementary Note 3

Given two corresponding point sets $X = \{x_1, \cdots, x_n\}$ and $P = \{p_1, \cdots, p_n\}$, we wish to obtain a rotation R and a translation t such that the mean squared error

$$E(r, t) = \frac{1}{N} \sum_{i=1}^{N} ||x_i - Rp_i - t||^2$$

is minimized. For generating the correct correspondences, we propose a heuristic approximation where the nearest neighbors in the point cloud represent corresponding synapses. The closest alignment is found iteratively and is said to converge if the error in spatial alignment of both clouds is within a specified tolerance value t_0.

Supplementary Note 4

More often than not, synapse point clouds of the fixed and moving image volumes are deformed by a non-rigid transformation. Generally, these points do not lie on a structured grid. This encourages the use of interpolants obtained from Radial Basis Functions (RBFs).

Thin-plate splines themselves a natural representation in the form of RBFs. For a set of control points $\{c_i, i = 1,2, \cdots, K\}$ a spatial mapping is defined from an arbitrary location x to a new location $f(x)$.

$$f(x) = \sum_{i=1}^{K} w_i\, \varphi(||x - c_i||)$$

$||\cdot||$ denoting the $L2$-norm of the vectors and $\{w_i\}$ is a set of mapping coefficients. The thin plate spline corresponds to the kernel given by

$$\varphi(r) = r^2 \log r$$

Supplementary References

1. Seabold, S. & Perktold, J. Statsmodels: Econometric and Statistical Modeling with Python. *PROC. 9th PYTHON Sci. CONF* (2010).
