# Supplementary material for: Multiplexed expansion revealing for imaging multiprotein nanostructures in healthy and diseased brain
Source: Nat Commun. 2024 Nov 9;15:9722. doi: 10.1038/s41467-024-53729-w (PMC11550395; doi:10.1038/s41467-024-53729-w)
Supplement: Supplementary file 2 — Reporting Summary [file 41467_2024_53729_MOESM2_ESM.pdf]

nature portfolio

Corresponding author(s): Ed Boyden

Last updated by author(s): 2024.7.22

Reporting Summary

Nature Portfolio wishes to improve the reproducibility of the work that we publish. This form provides structure for consistency and transparency in reporting. For further information on Nature Portfolio policies, see our Editorial Policies and the Editorial Policy Checklist.

Statistics

For all statistical analyses, confirm that the following items are present in the figure legend, table legend, main text, or Methods section.

n/a	Confirmed	
☐	☒	The exact sample size (*n*) for each experimental group/condition, given as a discrete number and unit of measurement
☐	☒	A statement on whether measurements were taken from distinct samples or whether the same sample was measured repeatedly
☐	☒	The statistical test(s) used AND whether they are one- or two-sided<br>*Only common tests should be described solely by name; describe more complex techniques in the Methods section.*
☐	☒	A description of all covariates tested
☐	☒	A description of any assumptions or corrections, such as tests of normality and adjustment for multiple comparisons
☐	☒	A full description of the statistical parameters including central tendency (e.g. means) or other basic estimates (e.g. regression coefficient) AND variation (e.g. standard deviation) or associated estimates of uncertainty (e.g. confidence intervals)
☐	☒	For null hypothesis testing, the test statistic (e.g. *F*, *t*, *r*) with confidence intervals, effect sizes, degrees of freedom and *P* value noted<br>*Give P values as exact values whenever suitable.*
☒	☐	For Bayesian analysis, information on the choice of priors and Markov chain Monte Carlo settings
☒	☐	For hierarchical and complex designs, identification of the appropriate level for tests and full reporting of outcomes
☒	☐	Estimates of effect sizes (e.g. Cohen's *d*, Pearson's *r*), indicating how they were calculated

Our web collection on statistics for biologists contains articles on many of the points above.

Software and code

Policy information about availability of computer code

Data collection	Raw image data were collected by a Nikon Eclipse Ti inverted microscope with NIS-Elements software. Preprocessed, registered data are available for download from Harvard Dataverse at https://dataverse.harvard.edu/privateurl.xhtml?token=3cd5c49f-12bc-4849-a016-8b925691e8c2; DOI: https://doi.org/10.7910/DVN/JJBULY. Processed data derivatives used to generate plots are provided as Source Data with the manuscript.
Data analysis	Fiji/ImageJ and custom MATLAB (versions R2020a-b, R2022a-b, and R2023a) scripts were used to process images. Python 3 was used for some statistical analyses. All custom image processing and analysis scripts for all analyses in this manuscript are available at https://github.com/schroeme/multi-ExR, https://github.com/dgoodwin208/ExSeqProcessing, and https://github.com/donglaiw/ExM-Toolbox/tree/ck/mExR.

For manuscripts utilizing custom algorithms or software that are central to the research but not yet described in published literature, software must be made available to editors and reviewers. We strongly encourage code deposition in a community repository (e.g. GitHub). See the Nature Portfolio guidelines for submitting code & software for further information.

Data

Policy information about availability of data

All manuscripts must include a data availability statement. This statement should provide the following information, where applicable:

- Accession codes, unique identifiers, or web links for publicly available datasets
- A description of any restrictions on data availability
- For clinical datasets or third party data, please ensure that the statement adheres to our policy

Data availability statement is available in the manuscript.

Research involving human participants, their data, or biological material

Policy information about studies with human participants or human data. See also policy information about sex, gender (identity/presentation), and sexual orientation and race, ethnicity and racism.

Reporting on sex and gender	Not applicable.
Reporting on race, ethnicity, or other socially relevant groupings	Not applicable.
Population characteristics	Not applicable.
Recruitment	Not applicable.
Ethics oversight	Not applicable.

Note that full information on the approval of the study protocol must also be provided in the manuscript.

Field-specific reporting

Please select the one below that is the best fit for your research. If you are not sure, read the appropriate sections before making your selection.

[x] Life sciences [ ] Behavioural & social sciences [ ] Ecological, evolutionary & environmental sciences

For a reference copy of the document with all sections, see nature.com/documents/nr-reporting-summary-flat.pdf

Life sciences study design

All studies must disclose on these points even when the disclosure is negative.

Sample size	Studies were done with 2 or more mouse specimens with 3 or more fields of view each.
Data exclusions	Some channels were excluded from analysis after imaging due to poor signal quality, as detailed in Supplementary Tables 4 and 9. These data are still provided for download from Harvard Dataverse. Some ROIs were excluded during image analysis due to visible offset after registration. Details and criteria for exclusion are detailed in the Methods section. As described in the text, for statistics describing registration quality quantification, outliers were removed using GraphPad Prism's ROUT method (Motulsky and Brown, BMC Bioinformatics 2006).
Replication	All attempts at replication were successful.
Randomization	Randomization is not relevant to this study because the synapse staining or validation dataset of the somatosensory cortex were obtained from one strain (C57BL/6), and the amyloid-beta staining was obtained from 5xFAD, with WT as a control.
Blinding	Blinding was not relevant to most of this study because<br>1. the synapse staining or validatin dataset of the somatosensory cortex were imaged as is, and analyzed after taking images.<br>2. there are very obvious differences in WT and 5xFAD tissues regarding the presence of amyloid plaques.

Reporting for specific materials, systems and methods

We require information from authors about some types of materials, experimental systems and methods used in many studies. Here, indicate whether each material, system or method listed is relevant to your study. If you are not sure if a list item applies to your research, read the appropriate section before selecting a response.

Materials & experimental systems

n/a	Involved in the study	
[ ]	[x]	Antibodies
[x]	[ ]	Eukaryotic cell lines
[x]	[ ]	Palaeontology and archaeology
[ ]	[x]	Animals and other organisms
[x]	[ ]	Clinical data
[x]	[ ]	Dual use research of concern
[x]	[ ]	Plants

Methods

n/a	Involved in the study	
[x]	[ ]	ChIP-seq
[x]	[ ]	Flow cytometry
[x]	[ ]	MRI-based neuroimaging

Antibodies

Antibodies used	The manuscript (see Supplementary Information) has antibody information including supplier names and catalog numbers.
Validation	All antibodies which were used in this study are commercially available. We used commercial antibodies that had at least one and often 2 or more of the following: knockout or knockdown validation in tissue or cell lines, high user rating on vendor websites, multiple references for publications using the antibody, and vendor-provided images of Western blots and/or immunohistochemistry performed with the antibody (Supplementary Table 18).

Animals and other research organisms

Policy information about studies involving animals; ARRIVE guidelines recommended for reporting animal research, and Sex and Gender in Research

Laboratory animals	Both male and female wild type mice (C57BL/6, 6-8 weeks) and 5xFAD and age-matched wild type mice (12-13 months) were used.
Wild animals	This study did not involve wild animals.
Reporting on sex	Both male and female mice were used.
Field-collected samples	This study did not involve samples collected from the field.
Ethics oversight	All procedures involving animals were in accordance with the US National Institutes of Health Guide for the Care and Use of Laboratory Animals and approved by the Massachusetts Institute of Technology Committee on Animal Care.

Note that full information on the approval of the study protocol must also be provided in the manuscript.
